# Case report: CD38-directed CAR-T cell therapy: A novel immunotherapy targeting CD38- positive blasts overcomes TKI and chemotherapy resistance of myeloid chronic myeloid leukemia in blastic phase

**DOI:** 10.3389/fimmu.2022.1012981

**Published:** 2022-11-29

**Authors:** Qingya Cui, Peiqi Liang, Haiping Dai, Wei Cui, Mengjie Cai, Zixuan Ding, Qinfen Ma, Jia Yin, Zheng Li, Sining Liu, Liqing Kang, Li Yao, Jiannong Cen, Hongjie Shen, Mingqing Zhu, Lei Yu, Depei Wu, Xiaowen Tang

**Affiliations:** ^1^ National Clinical Research Center for Hematologic Diseases, Jiangsu Institute of Hematology, The First Affiliated Hospital of Soochow University, Suzhou, Jiangsu, China; ^2^ Institute of Blood and Marrow Transplantation, Collaborative Innovation Center of Hematology, Soochow University, Suzhou, Jiangsu, China; ^3^ School of Chemistry and Molecular Engineering, East China Normal University, Shanghai, China; ^4^ Shanghai Unicar-Therapy Bio-Medicine Technology Co., Ltd. Shanghai, China

**Keywords:** chronic myeloid leukemia, blast phase, tyrosine kinase inhibitor, chimeric antigen receptor T cell, CD38

## Abstract

Resistance to tyrosine kinase inhibitor (TKI) is a tough problem in the treatment of chronic myeloid leukemia in blastic phase (CML-BP), which was often associated with acquired mutations in the kinase domain and not eliminating the leukemic stem cells. The efficacy of TKI or combination with chemotherapy in CML-BP remains unsatisfactory. Chimeric antigen receptor T (CAR-T) cell immunotherapy may overcome TKI and chemotherapy resistance. However, lack of ideal targetable antigens is a major obstacle for treating patients with myeloid malignancies. CD38 is known to be expressed on most (acute myeloid leukemia) AML cells, and its lack of expression on hematopoietic stem cells renders it as a potential therapeutic target for myeloid CML-BP. We develop a CD38-directed CAR-T cell therapy for AML, and two patients with myeloid CML-BP were enrolled (NCT04351022). Two patients, harboring *E255K* and *T315I* mutation in the *ABL* kinase domain, respectively, were resistant to multiple TKIs (imatinib, dasatinib, nilotinib, and ponatinib) and intensive chemotherapy. The blasts in the bone marrow of two patients exhibited high expression of CD38. After tumor reduction chemotherapy and lymphodepletion chemotherapy, 1 × 10^7^ CAR-T-38 cells per kilogram of body weight were administered. They achieved minimal residual disease–negative and *BCR::ABL1*-negative complete remission and experienced grade II cytokine release syndrome manifesting as fever. Our data highlighted that CAR-T-38 cell therapy may overcome TKI and chemotherapy resistance in patients with myeloid CML-BP.

## Introduction

Treatment of chronic myeloid leukemia (CML) with tyrosine kinase inhibitor (TKI) has substantially extended patient survival, but the long-term cumulative probability of progression from chronic phase (CP) to blast phase (BP) is about 5% in the era of TKI therapy (1, 2). CML-BP remains a challenging disease, and myeloid CML-BP appears to have an inferior survival compared with patients with lymphoid CML-BP (3, 4). Single-agent TKI provides only modest and short-lived response for advanced phase CML (5, 6). Furthermore, similar responses were reported in patients with imatinib-resistant CML-BP after the treatment with the second-generation TKIs (nilotinib and dasatinib) (6). TKIs combined with intensive chemotherapy followed by allo-hematopoietic stem cell transplantation (HSCT) have improved survival of patients with CML-BP compared with that of TKI alone (7, 8). Response to treatment is the most important prognostic factor for survival of CML-BP (9). Therefore, novel approaches are needed to reinsert a deep molecular response before HSCT, especially for patients with TKI and chemotherapy resistance.

Chimeric antigen receptor T (CAR-T) cell immunotherapy targeting and amplifying of immune killing may provide a potent therapy to eradicate TKI- and chemotherapy-resistant blasts. CD38 is known to be expressed on most AML blast cells or plasma cells in multiple myeloma but not on healthy hematopoietic stem cells (HSCs) (10). Our recent research showed that the CD38-directed CAR-T cell therapy had achieved a 66.7% overall remission rate in treating relapsed AML after HSCT (11). Therefore, the therapeutic strategy using CD38-directed CAR-T cells (CAR-T-38) targeting CD38-positive blasts to overcome TKI and chemotherapy resistance can be exploited therapeutically for myeloid CML-BP.

## Case presentation 1

**Figure 1:**
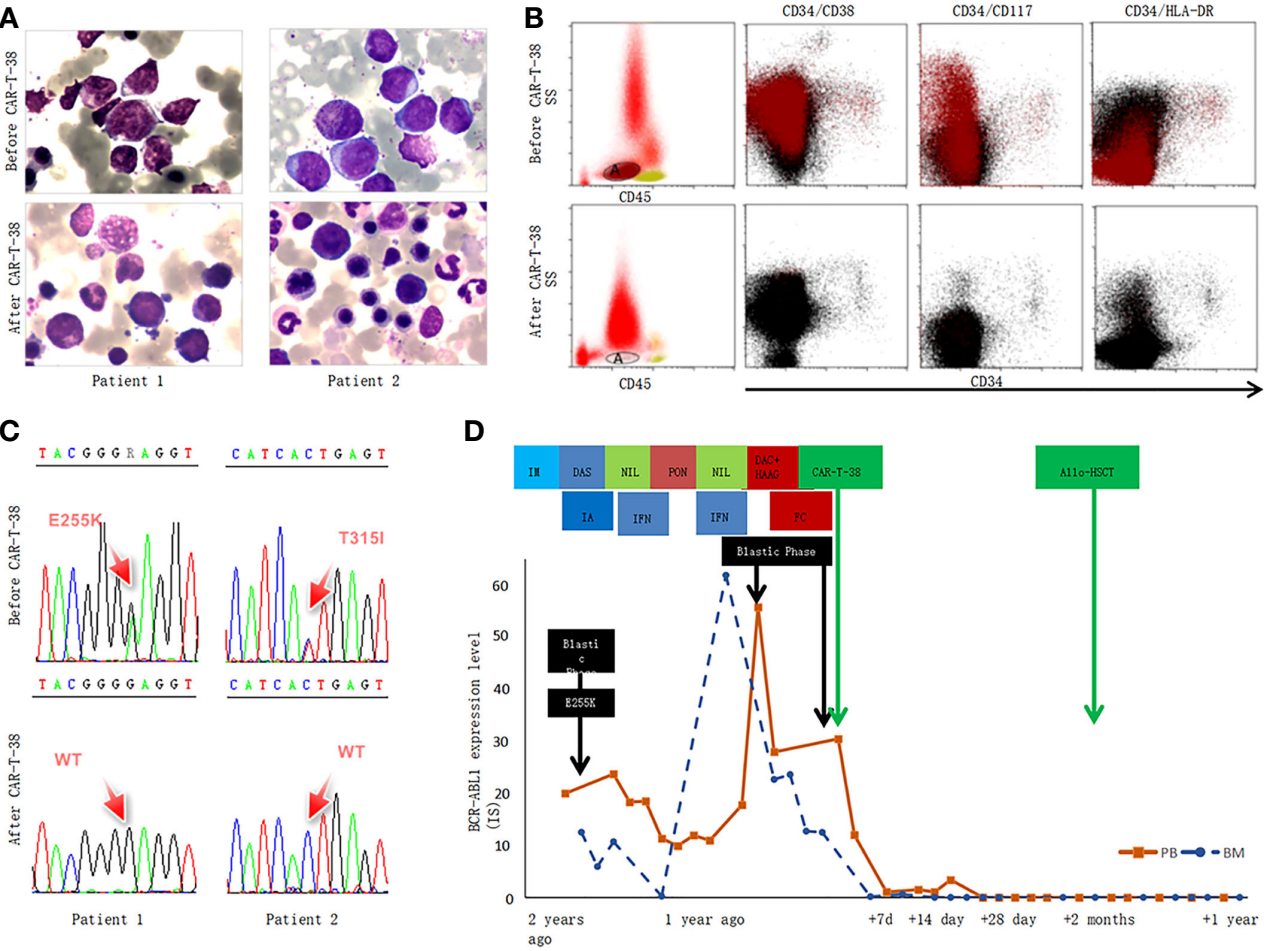
CAR-T-38 therapy response in the two patients with CML-BP. **(A)** This part showed the bone marrow morphological feature of patients 1 and 2 before (up) and 2 weeks after CAR-T-38 treatment (upper lower). After CAR-T-38 cell infusion, the patient 1 (left, lower) and 2 (right, lower) achieved CR. **(B)** The blasts were gated by using a CD45, side scatter (SS), and CD34-based gating strategy, but CD34^+^ leukemia had lost CD34 expression as the disease progressed to BP in these two patients, and the blasts were gated according to CD45/SS gating strategy (left). The blast (A population, tomato red) rates of bone marrow mononuclear cells (blank, middle and right plots) were 28.8% and 7.3 × 10^−4^ before and 2 weeks after CAR-T-38 treatment, respectively. This CD34^−^ AML blast fraction expressed CD38, CD117, CD33, CD13, CD123, and HLA-DR. **(C)** Both *E255K* and *T315I* mutation disappeared in bone marrow 2 weeks after CAR-T cell infusion. **(D)** Dynamic changes of *BCR::ABL1* expression level (IS) of patient 1 in the course of the disease. Patient 1 was resistant to multiple TKIs [imatinib (IM), dasatinib (DAS), nilotinib (NIL), and ponatinib (PON)], intensive chemotherapy with idarubicin and cytarabine (IA), and interferon-α (IFN). The level of *BCR::ABL1* was reduced to 0, 1 week after CAR-T treatment.

**Figure 2:**
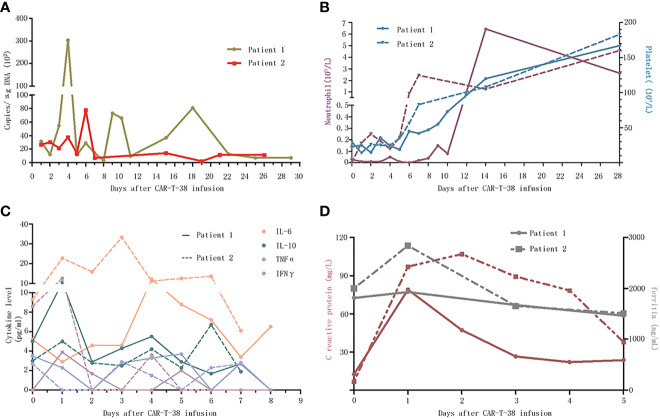
Dynamic changes after CAR-T-38 treatment. **(A)** The copies of CAR-T-38 cells in the peripheral blood measured by qPCR as the number of copies of lentiviral vector sequence per microgram of genomic DNA, which reached peak levels on days 4 and 6, respectively. **(B)** The neutrophil counts (left Y-axis) in patients 1 and 2 rose above 0.5 × 10^9^ on days 14 and 7, respectively, and platelet counts (right Y-axis) of the two patients rose above 100 × 10^9^ on day 14. **(C)** This part showed the changes of cytokines including IL-6, IL-10, TNFα, and IFNγ concentrations after CAR-T-38 infusion. **(D)** This part showed the changes of c-reaction protein (left Y-axis) and ferritin (right Y-axis) concentrations after CAR-T-38 infusion.

A 34-year-old male patient, complained with abdominal distension, was admitted to the hospital in January 2019. Blood routine showed a high white blood cell (WBC) count of 403 × 10^9^/L. Bone marrow (BM) examination suggested that CML in CP and flow cytometry (FCM) analysis showed 1.4% blasts with the expression of CD34^+^CD38^+^CD33^+^CD13^+^CD117^+^CD15^+^HLA-DR^+^CD36^+^ and MPO^+^. Philadelphia chromosome and *BCR::ABL1* fusion gene (P210) were detected. He was diagnosed as CML-CP with low risk (Sokal score of 0.37). The patient received imatinib (400 mg/day) and showed a significant decline in *BCR::ABL1* level. However, in July 2019, he presented blast crisis with 41% myeloid blasts in BM, and ABL1 kinase domain sequencing revealed P-loop (*E255K*) mutation. He received dasatinib (100 mg/day) combined with a standard “3 + 7” regimen with idarubicin and cytarabine (IA) and achieved a complete hematological response, and *BCR::ABL1^IS^* level was 12.5%. During follow-up on 15 November 2019, *BCR::ABL1^IS^* level increased to 23.7%, so he switched to nilotinib (400 mg, twice daily). One month later, *BCR::ABL1^IS^* level decreased to 11.30%, and interferon-α was given together with nilotinib, whereas on 30 December 2019, minimal residual disease (MRD) analysis showed 8.1% blasts, and *BCR::ABL1^IS^* was 10.9%. Considering the poor response to nilotinib, ponatinib (45 mg/day) was given instead. Half a month later, 39.9% blasts were detected in BM, and *BCR::ABL1^IS^* level increased to 55.7%. At this time, the immunophenotyping of CD34^−^CD38^+^CD117^+^CD33^+^CD13^+^CD123^+^HLA-DR^+^ was different to the onset of CML-CP. He was enrolled in a clinical trial of CAR-T-38 in relapsed or refractory AML (NCT04351022). Before CAR-T-38 cell infusion, decitabine (20 mg/m^2^/day, days 1–5) in combination with HAAG regimen (homoharringtonine, 1 mg/day, days 3–9; cytarabine, 10 mg/m^2^, injected subcutaneously every 12 hours, days 3–9; aclarubicin, 10 mg/day, days 3–6; granulocyte colony stimulating factor, 50–600 μg/day, days 2–9, unless WBC count was higher than 20 × 10^9^/L) was used to reduce tumor burden. The patient was pretreated with a fludarabine and cyclophosphamide (FC) regimen prior to CAR-T-38 immunotherapy. He showed no response to tumor-reduction chemotherapy and FC regimen. Then, CAR-T-38 cells, with a titer of 1 × 10^7^ cells per kilogram of body weight by dose escalation within 4 days, were administered. *In vivo*, CAR-T cell numbers increased rapidly and reached peak on day 4 (**Figure A**). He did not experience obvious adverse effects, manifesting as short-term myelosuppression stage (**Figure B**) and mild increase of cytokines and inflammatory mediators (**Figures 2C, D**). Two weeks after CAR-T-38 cell infusion, he achieved complete remission (CR) (**Figures 1A–C**). Moreover, *BCR::ABL1^IS^* decreased from 24% to 0% (**Figure 1D**). No off-target effect on monocytes and lymphocytes was observed (**Figure 1A, C**). To obtain longer survival, the patient received allo-HSCT from his brother at 7 weeks after CAR-T-38 cell therapy. Up to June 2022, he remained in complete hematologic reaction and complete molecular reaction (**Figure 1D**).

## Case presentation 2

A 32-year-old male patient was admitted to hospital for fatigue and fever in February 2018. Blood routine showed a high WBC count of 375×10^9^/L. A total of 4.5% blasts were detected in BM, and FCM analysis showed 4.9% blasts with CD34^+^CD33^+^CD13^+^CD11b^+^CD16^+^ and CD38^+^. Philadelphia chromosome and *BCR::ABL1* (P210) were positive. He was diagnosed as CML-CP with intermediate risk (Sokal score of 1.18), and imatinib (400 mg/day) was given to him. A month later, the *BCR::ABL1* level increased significantly, and *CBFβ/MYH11* fusion gene was detected. Then, he switched to dasatinib (100 mg/day). However, he was hospitalized again for fever in June 2018. The peripheral blood smear showed 40% blasts, and he received induction and consolidation chemotherapy with combination of dasatinib and standard “3 + 7” IA regimen. *T315I* mutation was detected during follow-up in December 2018, and then, he received ponatinib (45 mg/day) instead. Nevertheless, in March 2020, the BM smear exhibited 47% myeloid blasts, and immunophenotyping analysis showed 85.9% blasts with the expression of CD34^-^CD13^+^CD33^+^CD117^+^CD38^+^CD123^+^. RUNX1 and WT1 mutations were also detected. *BCR::ABL1^IS^* level increased to 21.4%. After enrollment in this clinical trial, he received the same treatment as patient 1 before CAR-T-38 cell infusion. The patient also showed no response to tumor-reduction chemotherapy and FC regimen. CAR-T cell numbers *in vivo* reached peak on day 6 (**Figure 2A**), and he did not have prominent adverse events (**Figures 2B–D**). One week after CAR-T-38 cell infusion, he achieved a CR (**Figure 1A**) and MRD-negative and *BCR::ABL1*-negative CR. Unfortunately, he was not able to receive allo-HSCT and died due to relapse and infection 2 months after CAR-T-38 cell infusion.

## Discussion

The majority of patients with CML-BP show myeloid phenotype; approximately 25% of patients with CML-BP show B lymphoid phenotype, and occasional patients transform to T lymphoid phenotype (12). As a myeloid malignant disease, although numerous tumor antigens, such as CD33, CD123, and CLL1, have been explored as potential target antigens for AML treatment in the past few decades, CAR-T cell therapy in AML remains challenging due to the lack of ideal antigen targets and the risk of fatal “off-tumor, on-target” side effects (13, 14). A few has been reported in CAR-T treatment of patients with myeloid CML-BP. CD38 is known to be expressed on most AML blast cells or plasma cells in multiple myeloma but not on healthy HSCs, which renders it as a potential therapeutic target for AML. CAR-T-38 cells have been evaluated mainly for their activity in multiple myeloma, and cytotoxicity against primary AML samples has also been confirmed (10, 15). Our recent research confirmed the clinical utility and safety of CAR-T-38 therapy for patients with AML with relapse after allo-HSCT (11). In the present research, blasts of these two patients exhibited over 95% expression of CD38. Two weeks after CAR-T-38 cell infusion, CD38-positive blasts were eradicated, and CD38-positive monocytes and lymphocytes were also reduced but recovered in a short time. The probable mechanism is that CAR-T-38 cells eliminated CD38-positive cells and not off-targeted on normal HSCs; subsequently, monocytes and lymphocytes can be differentiated form HSCs. At present, the molecular mechanisms of CML disease progression are still uncertain. Some findings lend support to the notion that *BCR::ABL1* oncogene arises in leukemia stem cells (LSCs), not yet committed to either myeloid or lymphoid differentiation (16). TKIs primarily target differentiated cells but cannot eliminate LSCs (17). Some attempts tried to develop immunotherapeutic strategies to target LSCs, such as CAR-T cells targeting CD123 and CLL-1 (18, 19). However, LSC phenotypes are usually heterogeneous. Recent studies find that a Lin^−^CD34^−^ fraction of CML-CP cells engrafted immunodeficient mouse strains, and CD34^+^ leukemia-initiating cells (LICs) had lost CD34 expression in mice (20). The population of CD34^−^CD38^+^ blasts of CML-CP likely contains the LICs. In these two patients, the blasts expressed CD34, CD38, CD33, CD13, CD117, and MPO in CP, whereas the immunophenotyping of blasts transformed to CD34^−^CD38^+^CD117^+^CD33^+^CD13^+^CD123^+^HLA-DR^+^ as the disease progressed to BP. After CAR-T-38 treatment, CD38-positive and *BCR::ABL1*-positive clones were both eliminated. We speculate that both *BCR::ABL1*-positive LSCs and myeloid differentiation with CD38 expression were eradicated by CAR-T-38 cells.

The immunosuppressive tumor microenvironment (TME) is a key factor limiting the applicability of CAR-T cells for tumors (21). CD38 is a glycoprotein that contributes to the tumorigenic properties of the TME. Recently, a study demonstrated that daratumumab treatment induces elimination of CD38-positive immune suppressor cells, such as regulatory T cells, regulatory B cells, and myeloid-derived suppressor cells (22). It is important to consider that not all off-target effects are undesirable and that elimination of CD38-positive immune suppressor cell subsets may lead to a beneficial therapeutic effect. Our CAR-T-38 with the same CD38 epitope (CD38 scFv) as daratumumab suggested that targeting CD38 may eliminate the CD38-positive blasts and restore immune suppressor TME. Cytokine release syndrome (CRS) and immune effector cell–associated neurotoxicity syndrome are well-known major adverse events that limit the clinical application of CAR-T cell therapy. In our research, two patients presented mild CRS (grade II), indicating it as a safe approach for patients with CML-BP.

TKI is considered as the cornerstone in the treatment of CML, and selection is of paramount importance. Ponatinib and asciminib are effective against *T315I* mutation (23, 24). Patient 2 in this study carried *T315I* mutation and progressed to blastic phase even if the ponatinib was used. Asciminib might be a good choice for patients with CML with *T315I* mutation. In recent years, some therapeutic options for acute myeloid leukemia are applied to CML-BP. TKIs, combined with hypomethylating agents, venetoclax, or both, have shown a promising efficacy (25). More treatment options for CML-BP should be explored.

In conclusion, our findings support the further clinical investigation of CAR-T-38 cells as a viable immunotherapeutic option to the treatment of patients with myeloid CML-BP who could be eligible for BM transplant.

## Data availability statement

The original contributions presented in the study are included in the article/Supplementary Material. Further inquiries can be directed to the corresponding authors.

## Ethics statement 

The studies involving human participants were reviewed and approved by the Ethics Committee of the First Affiliated Hospital of Soochow University. The patients/participants provided their written informed consent to participate in this study. Written informed consent was obtained from the individual(s) for the publication of any potentially identifiable images or data included in this article.

## Author contributions

DW, XT, LYu, HD, QC, PL, and SL were responsible for the study concept and design. QC and PL collected and analyzed the data and wrote the first draft of manuscript. XT, HD, JY, ZL, WC, and PL took care of these patients and assisted in data collection. QC, JC, ZD, QM, LK, HS, MC, and MZ provided input in the figures. XT, QC, PL, and HD wrote the final draft of manuscript. All authors read and approved the final manuscript.

## Funding

This work was supported by research grants from the National Natural Science Foundation of China (81873443, 82070162, 81900175, 81400155, 81700139, and 80900175), the Major Natural Science Research Projects in institutions of higher education of Jiangsu Province (19KJA210002), the Key Science Research Project of Jiangsu Commission of Health (K2019022), the Translational Research Grant of NCRCH (2020ZKZC04), the Natural Science Foundation of Jiangsu Province (BK20190181, BK20201169, BK20170360), the Frontier Clinical Technical Project of the Science and Technology Department of Jiangsu Province (BE2018652), the Priority Academic Program Development of Jiangsu Higher Education Institutions (PAPD), and the Natural Science Foundation of Jiangsu Province (BK2019042931).

## Conflict of interest

Authors LK and LYu were employed by Shanghai Unicar-Therapy Bio-Medicine Technology Co., Ltd.

The remaining authors declare that the research was conducted in the absence of any commercial or financial relationships that could be construed as a potential conflict of interest.

## Publisher’s note

All claims expressed in this article are solely those of the authors and do not necessarily represent those of their affiliated organizations, or those of the publisher, the editors and the reviewers. Any product that may be evaluated in this article, or claim that may be made by its manufacturer, is not guaranteed or endorsed by the publisher.
